# Supplementary material for: Stable chaos and delayed onset of statisticality in unimolecular dissociation reactions
Source: Commun Chem. 2020 Jan 8;3:4. doi: 10.1038/s42004-019-0252-y (PMC9814671; doi:10.1038/s42004-019-0252-y)
Supplement: Supplementary file 1 — Supplementary Information [file 42004_2019_252_MOESM1_ESM.pdf]

Supplementary information for: Stable chaos and delayed onset of statisticality in unimolecular dissociation reactions

Sourav Karmakar[1], Pankaj Kumar Yadav[1], and Srihari Keshavamurthy[1,*]

[1]Department of Chemistry, Indian Institute of Technology, Kanpur, Uttar Pradesh 208016, India

Contents

1 Supplementary Table 1. Parameters for Bunker's model # 6 **2**

2 Supplementary Methods **2**

2.1 Computation of the survival probabilities and lifetime distributions. 2

2.2 Multi-exponential fits to the survival probability. 3

2.3 Fast Lyapunov indicator and computation of the Arnold web. 4

2.4 Time-frequency analysis of the intramolecular dynamics. 7

3 Supplementary Note 1 **9**

4 Supplementary Note 2 **10**

5 Supplementary Note 3 **12**

6 Supplementary Note 4 **14**

7 Supplementary Fig. 9. Dependence of the Arnold web on θ_2 angle. **16**

8 Supplementary Fig. 10. Dependence of the Arnold web on θ_1 angle. **17**

9 Supplementary Note 5 **18**

10 Supplementary Note 6 **19**

11 Supplementary Fig. 13. Dependence of the Arnold web on energy. **21**

12 Supplementary Note 7 **22**

Supplementary References **24**

1 Supplementary Table 1. Parameters for Bunker's model # 6

Supplementary Table 1. Values of the potential parameters (in atomic units, except q_3^0) and G-matrix elements.

q_1^0	q_2^0	q_3^0 (radians)	α_1	α_2 *
2.416	2.416	2.039	2.212	2.069
$G_{11}^{(0)} = G_{22}^{(0)}$	$G_{33}^{(0)}$	$G_{12}^{(0)}$	$G_{13}^{(0)} = G_{23}^{(0)}$	
6.85×10^{-5}	2.88×10^{-5}	-1.54×10^{-5}	1.26×10^{-5}	

* The Morse exponents are chosen such that the harmonic frequencies corresponding to the stretching modes are 1112 and 1040 cm^{-1}.

2 Supplementary Methods

2.1 Computation of the survival probabilities and lifetime distributions.

For the survival probability and lifetime distribution computations (Fig. 1a of the main text), the phase space initial conditions for the trajectories at energy $E = 34$ kcal mol^{-1} are obtained as follows. The energetically accessible extent along the momentum direction is $p_i \in [-p_{i,\max}, p_{i,\max}]$, where $p_{i,\max} = (2E/G_{ii}^{(0)})^{1/2}$. Along q_i ($i = 1,2$) direction, $q_i \in [q_{i,\min}, q_{i,\max}]$, where $q_{i,\min}$ is obtained from the relation, $V_i(q_{i,\min}) = E$ and $q_{i,\max}$ is set to 5.5 au (considered maximum range). The allowed values for q_3 are then obtained from the constraint of energy conservation $H_\varepsilon(\mathbf{p}, q_1, q_2; q_3) = E$. For the survival probability computations shown in Fig. 1b in the main text, a slice of the allowed energy shell is sampled as follows. First we choose a specific angle section $\boldsymbol{\theta}_s \equiv (\theta_{1s}, \theta_{2s}, \theta_{3s})$. The allowed range of J_i ($i = 1,2$) is, $J_i \in [0, J_{i,\max}]$, where $J_{i,\max} = 2D_i/\omega_i$. The allowed values for J_3 are then obtained from $H_\varepsilon(\boldsymbol{\theta}_s, J_1, J_2; J_3) = E$.

Suitably large grids along each of the phase space variables ($\{\mathbf{q}, \mathbf{p}\}$ or $\{\boldsymbol{\theta}, \mathbf{J}\}$) are placed and the energy shell is sampled uniformly. The grid sizes $N_{q,p_{1,2}}$ along q_i and p_i ($i = 1,2$) are chosen to be the same and along p_3 it is set to N_{p_3}. For the ensemble used in the results shown in the main text (Fig. 1a), $(N_{q,p_{1,2}}, N_{p_3}) = (20, 28)$. For Fig. 1b in the main text, the grid sizes are $(N_{J_1}, N_{J_2}) = (500, 500)$. The size of different ensembles for different ε's used in the main text for survival probability computation is listed in Supplementary Table 2. In addition, Supplementary Table 2 also provides information on the number of trajectories analyzed using wavelet time-frequency analysis (Fig. 4 of the main text). The initial conditions were then used to integrate the Hamilton's equations of motion for $T = 40$ ps using a 8th order Runge-Kutta method[1]. The total energy of the trajectories were found to be conserved with sufficient accuracy. For example, the energy of a typical non-dissociated highly chaotic trajectory is conserved up to 8th decimal place for the entire duration of the integration.

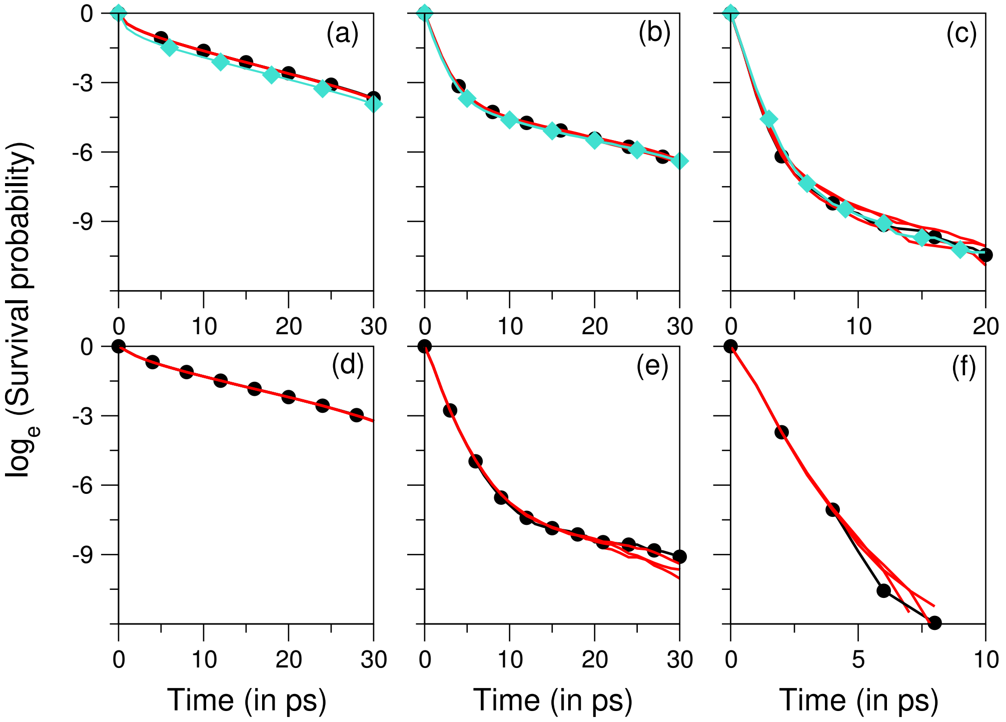

Supplementary Figure 1. Convergence studies of survival probability for different ε in Fig. 1a (upper panel) and Fig. 1b (lower panel) of the main text. (a), (d) $\varepsilon = 0.1$, (b), (e) $\varepsilon = 0.4$, and (c), (f) $\varepsilon = 1.0$. Four different grid sizes used in the upper panel are $(N_{q,p_{1,2}}, N_{p_3}) = (19,27)$, $(20,28)$, $(21,29)$ and $(22,30)$. For the lower panel the grid sizes are $(N_{J_1}, N_{J_2}) = (400,400)$, $(500,500)$, $(600,600)$ and $(700,700)$. In both the panels the ensemble shown in black circles is used in the main text and the results for the other grid sizes are shown in red. The survival probabilities in the top panel, shown in cyan (diamonds), corresponds to setting $q_{i,\max} = 7.5$ au with $(N_{q,p_{1,2}}, N_{p_3}) = (24,32)$.

The size of the grids were varied to create different ensemble sizes to check for convergence. Supplementary Fig. 1 shows the survival probability to be nearly converged. Slight variations at long times and large values of ε are due to the very small number of surviving trajectories. Note that the value of $q_{i,\max}$ up to 7.5 au has been considered in order to check for convergence of the survival probabilities. The survival probabilities, as shown in Supplementary Fig. 1 (cyan diamonds), show slightly faster decay at initial times for small ε. However, the differences at later times are not significant. Moreover, the slopes of the survival probabilities do not depend much on the chosen value of $q_{i,\max}$. Therefore, for all the results shown in this work, the value of $q_{i,\max}$ is set to 5.5 au.

2.2 Multi-exponential fits to the survival probability.

The non-exponential behaviour of the survival probabilities in Fig. 1 in the main text are fit to a general bi-exponential function[2] of the form,

$$S(\tau) = \frac{N(\tau)}{N(0)} = f_1 e^{-k_1 \tau} + f_2 e^{-k_2 \tau} \tag{1}$$

Supplementary Table 2. Total number of trajectories used for computing the lifetime distributions and survival probabilities (Fig. 1 of the main text).

ε	0.1	0.2	0.3	0.4	1.0
total # of trajectories *	180458	181380	182442	183878	207706
# of trajectories with lifetime $t_{lt} \in (3.5, 4.5)$	6778	9298	5957	3332	237
# of undissociated trajectories	36169	15571	7832	3775	48
total # of trajectories **	137847	139238	140811	142518	155286
# of trajectories with lifetime $t_{lt} \in (3.5, 4.5)$	6916	9952	6373	3130	200
# of undissociated trajectories	26845	5413	793	22	0

* For Fig. 1a. in the main text
** For Fig. 1b. in the main text

where $\sum_{i=1}^{2} f_i = 1$ due to normalization. $N(\tau)$ is the number of undissociated trajectories at $t = \tau$ and $N(0)$ is the size of the dissociating ensemble i.e., the total number of trajectories which dissociate within the integration time period of $T = 40$ ps. We have used MATLAB[3] to fit the numerical data up to 30 ps.

The resulting fits for different ε values are shown in Supplementary Table 3. In the table we also show the result of the short time (1 ps) single exponential fits for the survival probabilities shown in Fig. 1a of the main text. In Supplementary Table 4 the values of the fit parameters for Fig. 1b of the main text are shown. Note that in both cases a single exponential with rate constant $k_1 \sim 1.7$ ps^{-1} is sufficient for $\varepsilon = 1.0$.

The accuracy of the fits are indicated in Supplementary Fig. 2a-b in terms of the residual defined as

$$\text{Residual} = \left| \frac{\ln S(\tau) - \ln \tilde{S}(\tau)}{\ln S(\tau)} \right| \times 100 \quad (2)$$

where $\mathrm{S}(\tau)$ is computed from the original lifetime data set and $\tilde{\mathrm{S}}(\tau)$ corresponds to the fitted data. Notice that in Supplementary Fig. 2a, the residual is very large for $\varepsilon = 1.0$ at longer times. This is expected since the fit is to a single exponential form and the number of trajectories contributing to the origin of the second time scale is extremely small.

2.3 Fast Lyapunov indicator and computation of the Arnold web.

There are several methods to compute the Arnold web. All of these methods involve integrating additional differential equations to determine appropriate variational chaos indicators over short timescales. A comprehensive account of the various indicators can be found in a recent compendium of articles[4]. In this work we have used the relatively less expensive fast Lyapunov

Supplementary Table 3. Results of the fit to the survival probabilities in Fig. 1a of the main text.

ε	f_1	k_1 (ps^{-1})	f_2	k_2 (ps^{-1})	k (ps^{-1}) **
0.1	0.466	1.227	0.533	0.101	0.605
0.2	0.882	0.528	0.118	0.106	0.624
0.3	0.964	0.738	0.036	0.092	0.844
0.4	0.972	1.025	0.027	0.089	1.125
1.0 *	0.999	1.705	–	–	1.943

* Single exponential fit.
** Single exponential fit based on 1 ps data.

Supplementary Table 4. Results of the fit to the survival probabilities in Fig. 1b of the main text.

ε	f_1	k_1 (ps^{-1})	f_2	k_2 (ps^{-1})
0.1	0.296	0.643	0.703	0.093
0.2	0.816	0.426	0.184	0.119
0.3	0.982	0.615	0.018	0.098
0.4	0.997	0.851	0.002	0.125
1.0 *	1.000	1.712	–	–

* Single exponential fit.

indicator (FLI) method. The FLI has been used to map the Arnold web for various dynamical systems and details can be found in the literature[5,6]. Here we provide a brief introduction to computing the FLI.

For a general dynamical system,

$$\frac{d\mathbf{X}}{dt} = \mathbf{F}(\mathbf{X}) \tag{3}$$

with $\mathbf{X} = \{x_1, x_2, \ldots, x_i, \ldots\}$ being the dynamical variables, the FLI[7] is defined as

$$\mathrm{FLI}(\mathbf{X}(0), \mathbf{v}(0), t) = \log||\mathbf{v}(t)|| \tag{4}$$

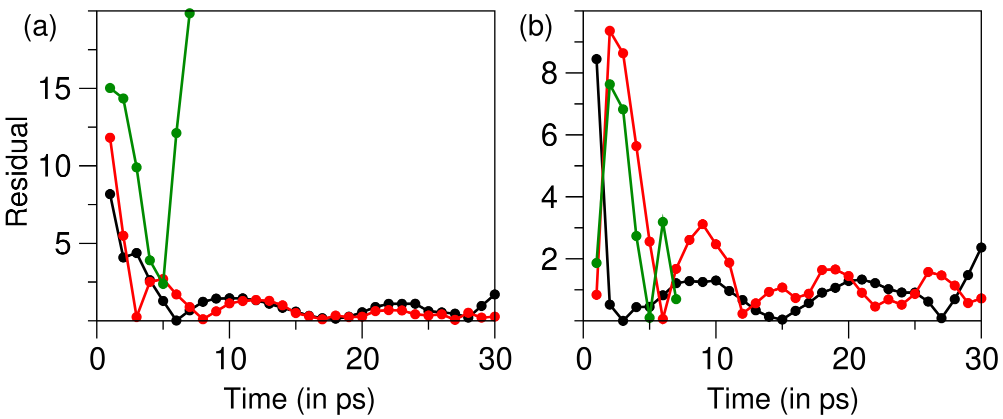

Supplementary Figure 2. Residual data of the lifetime distribution fitting shown in the main text (Fig. 1a-b respectively) for $\varepsilon = 0.1$ (black), 0.4 (red), 1.0 (green).

where, $\mathbf{v}$ is a tangent vector and

$$\frac{d\mathbf{v}}{dt} = \left(\frac{\partial \mathbf{F}}{\partial \mathbf{X}}\right)\mathbf{v} \tag{5}$$

However for technical reasons the alternative definition[5]

$$\mathrm{FLI}(\mathbf{X}(0), \mathbf{v}(0), t) = \sup_{0<t'<t} \log||\mathbf{v}(t')|| \tag{6}$$

is usually preferred. Consequently, in this work we use Supplementary Equation 6 to compute the FLI. The variation of FLI with time for regular and chaotic trajectories is distinctly different and hence different types of dynamical behaviour can be identified, usually within timescales t_f much shorter compared to those required for computing the Lyapunov exponents.

In Supplementary Fig. 3 we show an example for the behaviour of FLI for different types of trajectories. For regular trajectories, the FLI saturates quickly whereas for the chaotic trajectory the FLI increases linearly. As can be seen from Supplementary Fig. 3, the distinction between chaotic and regular trajectories is already clear by ~ 10 ps. For comparison, in Supplementary Fig. 3b-d we also show the variations in the mode frequencies with time for the three different trajectories using wavelet-based time-frequency analysis (see below). For the regular trajectories, the mode frequencies are nearly constant. In contrast, for the chaotic trajectory the mode frequencies exhibit drifts and rapid fluctuations. Note that even for the chaotic trajectory the frequency of the bending mode, being modeled as a harmonic oscillator, does not fluctuate as much as the stretching frequencies.

In this work we compute the Arnold web in the action space as follows. For every initial condition on a specific slice of the action-angle space, as described in the previous section, we determine the associated FLI value using Supplementary Equation 6 with a final time of $T = 40$ ps. However, as discussed below, for trajectories that dissociate before $T = 40$ ps the total integration time T is set by the lifetime t_{lt} and hence the FLI value is noted at t_{lt}. Note that an arbitrary choice of the initial tangent vector can lead to spurious structures[8] on the web. To avoid this, we follow the prescription by Barrio et al[8] and choose the initial tangent vector as $-\nabla H/||\nabla H||$. In Supplementary Fig. 4 we show the advantage of using Supplementary Equation 6 for computing the web. The FLI defined in Supplementary Equation 4 leads to oscillatory

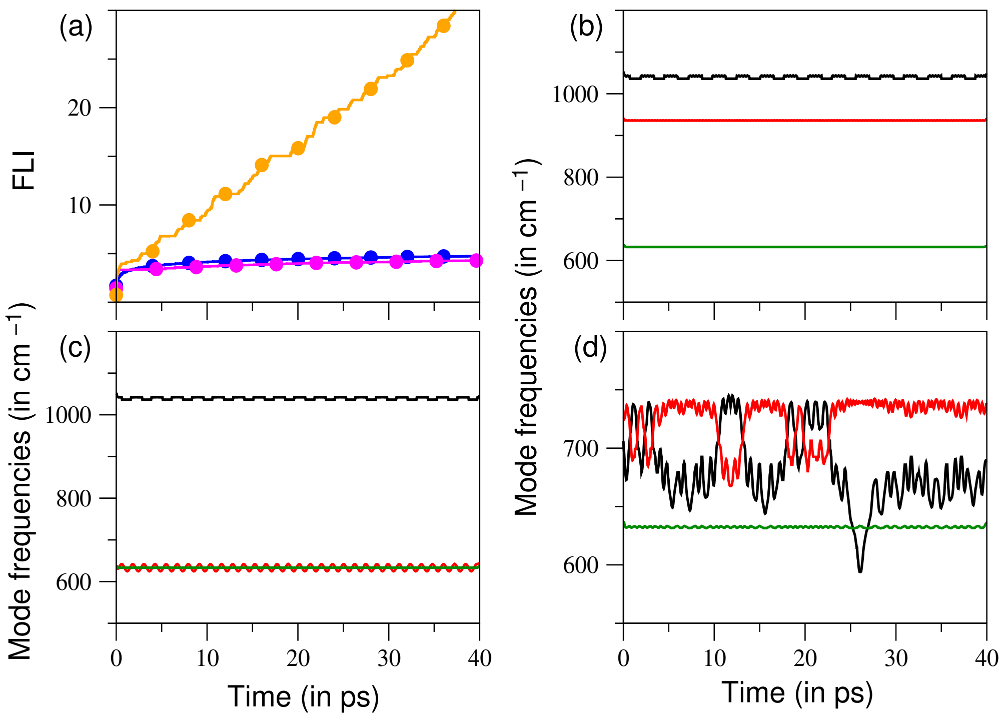

Supplementary Figure 3. Nature of FLI for different trajectories at $E = 34$ kcal mol^{-1} and $\varepsilon = 0.01$. (a) FLI for three different trajectories, non-resonant (blue), resonant (magenta) and chaotic trajectory (orange). Notice already by $T = 10$ ps, FLI is very different for regular and chaotic trajectory. (b), (c) and (d) are the mode frequencies of non-resonant, resonant and chaotic trajectory respectively (same as those shown in (a)) obtained from wavelet analysis (discussed later). Frequencies of the first stretching, second stretching and the bending mode are shown in black, red and green respectively.

features seen in Supplementary Fig. 4a. However, use of Supplementary Equation 6 does not have such features.

2.4 Time-frequency analysis of the intramolecular dynamics.

To compute the local frequencies we use the wavelet-based time-frequency approach[9] of Arevalo and Wiggins. In this approach local frequencies are determined by a continuous wavelet transform of the time series corresponding to the trajectories. Specifically, the wavelet transform provides the joint time-frequency information of a signal or time series. For a system in the near-integrable regime, according to the Kolmogorov-Arnold-Moser (KAM) theory, almost all of the unperturbed tori remain intact and the frequencies associated with the KAM tori can be approximated by $\mathbf{\Omega}^0(\mathbf{J}) \equiv \partial H_0(\mathbf{J})/\partial \mathbf{J}$. However with increasing the perturbation, the KAM tori are destroyed and both the regular and chaotic regions coexist in the phase space. In such cases, the frequencies associated with a chaotic trajectory can not be approximated by $\Omega \approx \Omega^0$. Although, for a chaotic trajectory there are several frequency components (Supplementary Fig. 11), the joint time-frequency analysis can be used to gain information on the dominant frequencies in an appropriate time-window. Several studies[9–11] have established that the dominant frequencies can be associated with the chaotic trajectory visiting specific set of phase space structures. In the molecular context, the dominant frequencies can be correlated to specific intramolecular motions

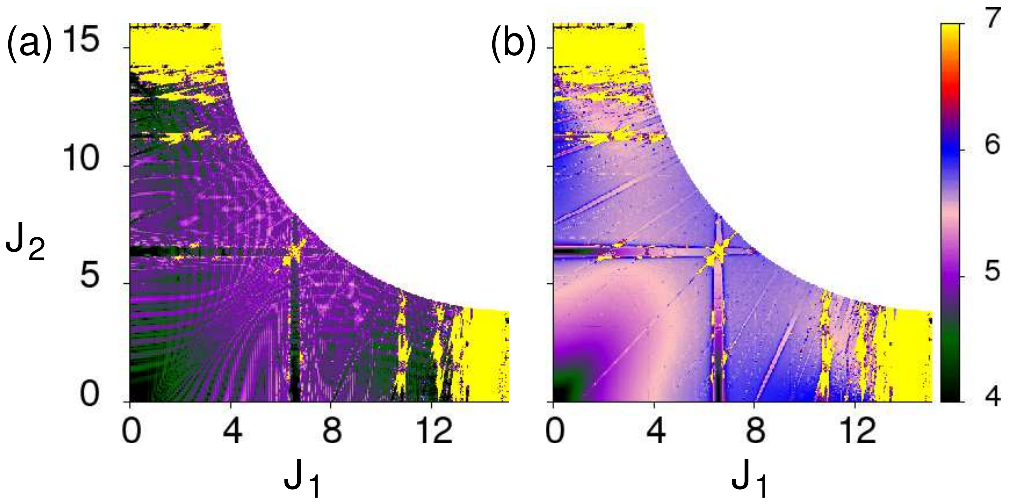

Supplementary Figure 4. Comparing the Arnold webs for $\varepsilon = 10^{-3}$ determined using the different FLI definitions. (a) using Supplementary Equation 4 (b) using Supplementary Equation 6.

with certain vibrational modes being more active than others in a given time interval. We give here a brief description of the time-frequency analysis since details can be found in previous works[9,12].

The time series $z_k(t) = q_k(t) + ip_k(t)$, obtained from the dynamics, for the three modes ($k = 1,2,3$) are subjected to a continuous wavelet transform

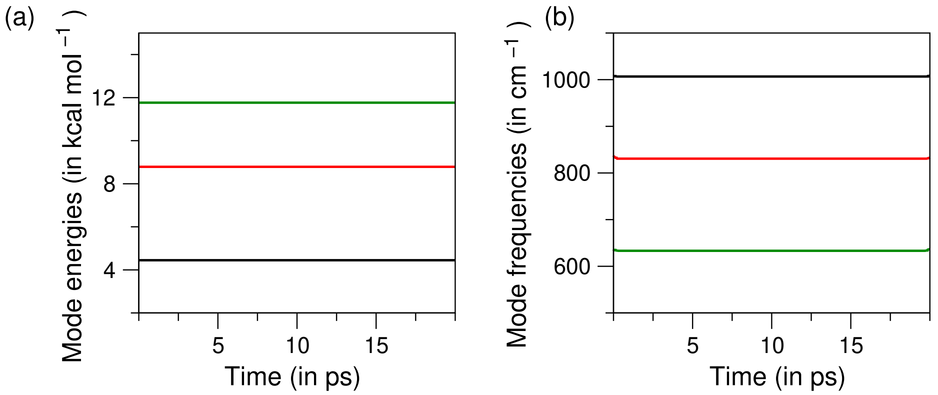

Supplementary Figure 5. (a) Mode energy versus time plot at energy $E = 25$ kcal mol^{-1} when all the modes are decoupled. Black, red, green lines represent the first stretch mode, second stretch mode and the bend mode respectively. (b) Mode frequency versus time for the trajectories obtained using continuous wavelet transform. Color code for different modes is the same.

$$L_\psi z_k(a,b) = a^{-1/2} \int_{-\infty}^{\infty} z_k(t) \psi^* \left(\frac{t-b}{a} \right) dt \tag{7}$$

where, $a > 0$ and b is real and

$$\psi(t) = \frac{1}{\sigma\sqrt{2\pi}} e^{2\pi i \lambda t} e^{-t^2/2\sigma^2} \tag{8}$$

is the Morlet-Grossman mother wavelet. λ and σ are the parameters which can be tuned for better accuracy. For our calculation, we have used $\lambda = 1$ and $\sigma = 2$. Other values for these parameters yield similar results with slightly differing accuracy, depending on whether the mode of interest is a low or high frequency one. Using Supplementary Equation 7 the local frequency associated with $z_k(t)$ over a small interval of time around $t = b$, related to the inverse of the scale factor a, can be determined. There are two ways of analyzing the transform. First is to extract the dominant frequency at a given time by computing the maximum of the modulus of the wavelet transform i.e., $\max_a |L_\psi \mathbf{z}(a,b)|$. The ratios of the dominant frequencies can then be used to analyze the dynamics in the frequency ratio space (FRS). A second more comprehensive way[11] is to compute the full scalogram $|L_\psi z_k(a,b)|^2$ to look at the ridge maxima and dominant frequency variations over time. Although we have examined the scalograms of several individual trajectories, in this work we have considered only the approach based on the dominant frequencies. The reason being that working with the scalogram for the large ensemble sizes considered in this work is rather impractical.

As a check for the accuracy of the wavelet transform, Supplementary Fig. 5 shows a representative example in the case when the system is uncoupled and the total energy of $E = 25$ kcal mol^{-1} is partitioned into the three modes. The energy of the each mode as a function of time is shown in Supplementary Fig. 5a. Note that due to the modes being uncoupled, there is no IVR and the individual mode energies are conserved. The frequencies computed using the wavelet analysis for this example are shown in Supplementary Fig. 5b. The calculated frequencies, using Supplementary Equation 17, of the Morse oscillators are 1003.9 cm^{-1} and 828.5 cm^{-1} respectively, which are very close to the numerically computed frequencies (1006.7 cm^{-1}, 830.6 cm^{-1}). In the presence of the resonant coupling terms, the system becomes non-integrable and the frequencies show nontrivial variations with time. The frequency variation depends on the nature of the trajectory. In Supplementary Fig. 3d and Supplementary Fig. 11, examples for such frequency variations can be seen.

3 Supplementary Note 1

A complication in interpreting the FLI results for unbounded systems, as studied in the present work, is worth noting at this point. For bounded systems a fixed, appropriately chosen time T is sufficient to discriminate between different types of dynamics. However, in our case the trajectories can dissociate with different lifetimes $t_{\rm lt}$. Thus, for trajectories with very short ($\ll 5$ ps) lifetimes the FLI values are expected to be small and hence indistinguishable from regular trajectories. In other words, the dissociation event sets the total integration timescale for each trajectory. However, note that such ambiguities occur only for trajectories with rather short lifetimes. As examples for such behaviour, in Supplementary Fig. 7 we show the comparison of FLI for different trajectories with varying lifetimes. For trajectories with small $t_{\rm lt}$ (Supplementary Fig. 7a), the FLI is very close to that of a regular trajectory. However, the value of $t_{\rm lt}$ helps in this regard to distinguish these two class of trajectories because of very large $t_{\rm lt}$ for regular trajectories. For relatively long $t_{\rm lt}$ (Supplementary Fig. 7b), the FLI is clearly capable of distinguishing between the different dynamical behaviours.

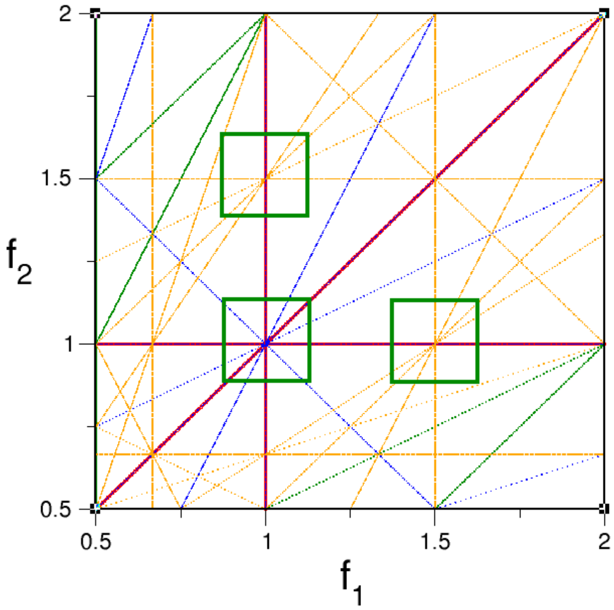

Supplementary Figure 6. Frequency ratio space $(f_1, f_2) = (\Omega_1/\Omega_3, \Omega_2/\Omega_3)$ for the full three degrees of freedom system showing the possible resonances up to order five. A few key resonance junctions are highlighted with green boxes.

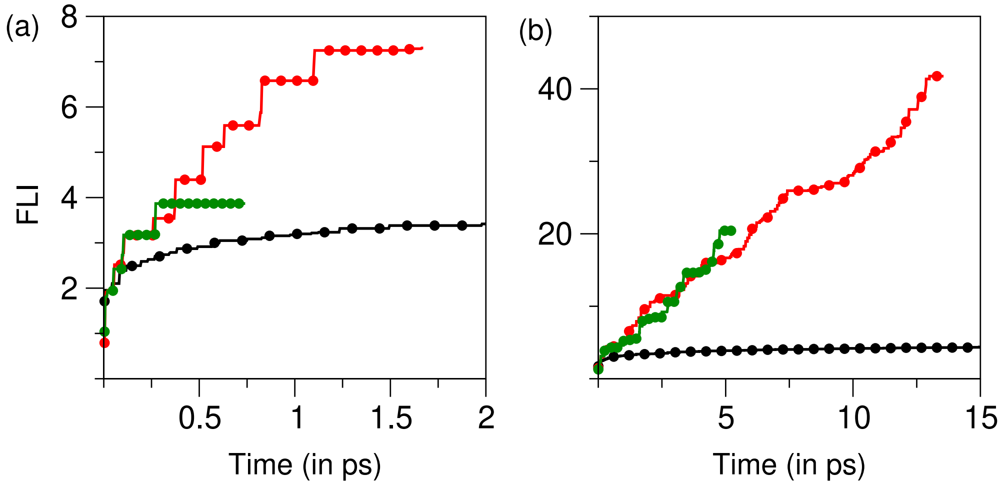

Supplementary Figure 7. Comparison of the FLI for trajectories with small and large dissociation lifetimes t_{lt} with a typical regular trajectory (undissociated till 40 ps, shown in black). (a) Trajectories with small t_{lt} (~ 0.75 ps and ~ 1.7 ps) (b) Trajectories with large t_{lt} (~ 5 ps and ~ 13 ps).

4 Supplementary Note 2

Here we provide a summary of the action-angle transformations and strengths of the nonlinear resonances extracted from the analysis.

For the bending mode the harmonic oscillator Hamiltonian used in the main text is of the

form

$$H_3^{(0)} = \frac{1}{2}G_{33}^{(0)}p_3^2 + \frac{1}{2G_{33}^{(0)}}\omega_3^2(q_3 - q_3^0)^2 \tag{9}$$

where, q_3 and p_3 are the conjugate coordinate and momentum and ω_3 is the frequency. The action-angle transformation is given by,

$$q_3 - q_3^0 = \sqrt{\frac{2G_{33}^{(0)}J_3}{\omega_3}}\sin\theta_3 \tag{10}$$

$$p_3 = \sqrt{\frac{2\omega_3 J_3}{G_{33}^{(0)}}}\cos\theta_3 \tag{11}$$

where J_3 and θ_3 are the action and angle variables respectively. In the action-angle representation the transformed Hamiltonian can be expressed as

$$H_3^{(0)}(J_3) = \omega_3 J_3 \tag{12}$$

As expected, the frequency $\partial H_3^{(0)}(J_3)/\partial J_3 = \omega_3$ is independent of the energy.

For the two stretching modes the Morse oscillator Hamiltonian is of the form

$$H_i^{(0)} = \frac{1}{2}G_{ii}^{(0)}p_i^2 + D_i\left(1 - e^{-\alpha_i(q_i - q_i^0)}\right)^2 \tag{13}$$

with α_i and D_i $(i = 1,2)$ being the Morse length scale parameter and the dissociation energy respectively. The action-angle transformation[13] can be expressed as

$$q_i - q_i^0 = \alpha_i^{-1}\ln[(1 + b_i\cos\theta_i)/\lambda_i^2] \tag{14}$$

$$p_i = \frac{\omega_i\lambda_i b_i\sin\theta_i}{G_{ii}^{(0)}\alpha_i(1 + b_i\cos\theta_i)} \tag{15}$$

Here, $\lambda_i(J_i) \equiv 1 - (\omega_i J_i/2D_i)$ and $b_i(J_i) \equiv \sqrt{E_i/D_i}$ are the parameters related to the energy $E_i = H_i$, harmonic frequency $\omega_i = \alpha_i\sqrt{2G_{ii}^{(0)}D_i}$ and the action variable J_i.

In the action-angle representation the Morse Hamiltonian transforms to

$$H_i^{(0)}(J_i) = \omega_i\left[J_i - \frac{\omega_i}{4D_i}J_i^2\right] \tag{16}$$

with the energy dependent nonlinear frequency

$$\Omega_i^{(0)}(J_i) \equiv \frac{\partial H_i^{(0)}(J_i)}{\partial J_i} = \omega_i\left(1 - \frac{\omega_i}{2D_i}J_i\right) \tag{17}$$

Based on the transformations above, the Hamiltonian in the main text (eq. 3,4), $H(\mathbf{J},\theta) \equiv H_0(\mathbf{J}) + \varepsilon V(\mathbf{J},\boldsymbol{\theta})$ has an integrable zeroth-order part

$$H_0(\mathbf{J}) = \omega_3 J_3 + \sum_{k=1,2} \omega_k \left[J_k - \frac{\omega_k}{4D_k} J_k^2 \right] \tag{18}$$

and the coupling term which, using the known Fourier expansion[13] of the momentum $p_j = \sum_{k=1}^{\infty} B_k^{(j)}(J_j)\sin(k\theta_j)$, can be explicitly written as a Fourier expansion

$$\begin{aligned} V(\mathbf{J},\boldsymbol{\theta}) &= \sum_{l,k=1}^{\infty} f_{l,k}^{(12)}(J_1,J_2)[\cos(l\theta_1 - k\theta_2) - \cos(l\theta_1 + k\theta_2)] \\ &\quad + \sum_{l=1}^{\infty} g_l^{(13)}(J_1,J_3)[\sin(l\theta_1 - \theta_3) + \sin(l\theta_1 + \theta_3)] \\ &\quad + \sum_{k=1}^{\infty} g_k^{(23)}(J_2,J_3)[\sin(k\theta_2 - \theta_3) + \sin(k\theta_2 + \theta_3)] \end{aligned} \tag{19}$$

where we have denoted

$$B_k^{(j)}(J_j) = (-1)^k \left(\frac{2\omega_j \lambda_j}{\alpha_j G_{jj}^{(0)}} \right) \left[\frac{1-\lambda_j}{1+\lambda_j} \right]^{k/2} \tag{20}$$

The coefficients in Supplementary Equation 19 can now be explicitly expressed as

$$f_{l,k}^{(12)}(J_1,J_2) = \frac{1}{2} G_{12}^{(0)} B_l^{(1)}(J_1) B_k^{(2)}(J_2) \tag{21}$$

$$g_l^{(i3)}(J_i,J_3) = \frac{1}{2} G_{i3}^{(0)} B_l^{(i)}(J_i) B^{(3)}(J_3) \tag{22}$$

for $i = 1,2$ and $B^{(3)}(J_3) = \sqrt{2\omega_3 J_3 / G_{33}^{(0)}}$.

5 Supplementary Note 3

Another way of visualizing a subspace of the phase space is by constructing the zero momentum surface (ZMS)[14]. The ZMS is defined as the surface with $p_i = 0$, for all i. To map the ZMS, a 500×500 grid is constructed along $q_1 - q_2$ keeping $p_1 = p_2 = p_3 = 0$. The allowed value of q_3 is then obtained from energy conservation. All the trajectories with initial conditions on the ZMS are integrated for 40 ps, using the same integrator mentioned above, and the resulting FLI is projected on the $q_1 - q_2$ plane.

In Supplementary Fig. 8 we show the ZMS for varying ε and the corresponding lifetime plots. Note that the different regular regions in the ZMS are clearly identified. Specifically, on comparing to the Arnold webs shown in Fig. 2 of the main text, specific structures on the ZMS can be related to the different resonance and the junctions formed by them. The corresponding lifetime plots also shows one-to-one correspondence with the ZMS features.

Note that the results in Supplementary Fig. 8 indicates persistence of regular regions near the potential minima with undissociated trajectories up until $\varepsilon \sim 1$. However, the survival probability results shown in Fig. 1 of the main text do not include such initial conditions. Thus,

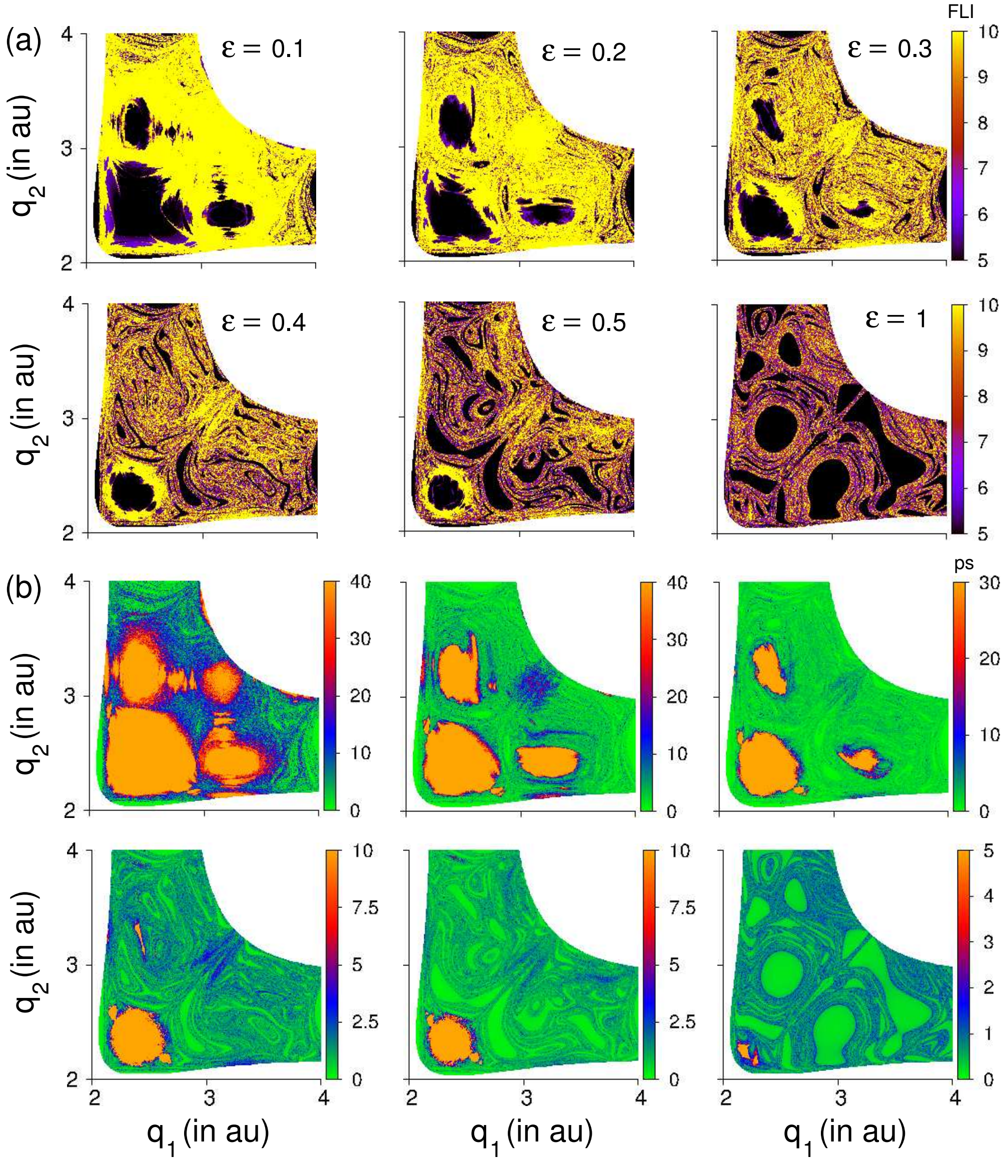

Supplementary Figure 8. (a) FLI corresponding to the ZMS, (b) lifetime (in picoseconds) of trajectories with initial conditions on the ZMS with varying values of ε.

the second longer timescales in the survival probabilities are not due to these regions. Instead, the long lifetime trajectories observed near the periphery of these regions and the partially broken 1:1 resonance regions give rise to the tail in the lifetime distributions. More importantly, and related to the results shown in Fig. 4 of the main text, the observation of significantly large lifetimes near the $\mathscr{M}^{1,0,-1}_{0,1,-1}$ junction regions at $q_1 \approx q_2 \sim 3.1$ in Supplementary Fig. 8 are key to the non-statisticality. We also point out that the regular region seen at $(q_1, q_2) \approx (2.1, 3.2)$ in Supplementary Fig. 8 for $\varepsilon = 0.4$ does not manifest on the Arnold web slice shown in Fig. 2 of the main text. Clearly, a combination of the (J_1, J_2) Arnold web slice and the ZMS yields fairly detailed information regarding the phase space structures that lead to strongly correlated intramolecular dynamics.

6 Supplementary Note 4

Here we briefly sketch possible estimates of the critical ε for the onset of widespread chaos using the idea based on the Chirikov criterion[15].

Thinking along the lines of Oxtoby and Rice[16], one can estimate the onset of extensive overlap and hence RRKM behavior can be predicted from the Arnold webs shown in the main paper. Note that a straightforward generalization of the overlap criterion to the present case is not obvious. In $f = 2$ the zeroth order energy surface is a line in the (J_1, J_2) action space. The various resonances puncture this zeroth order energy surface and the size of the holes created scales with the coupling strength. In our case the zeroth-order energy surface is in the (J_1, J_2) plane with the various resonances forming a dense network. Thus, it is not immediately apparent as to what set of resonances need to be considered for the estimate of critical ε. A natural choice would be the resonances of lowest order, since they have the largest widths. Still, which set of lowest order resonances is far from obvious. In the following, we use the fact that our computations indicate the 1:1 stretch-bend resonances as the prominent ones to consider.

We now sketch a calculation to obtain an approximate estimate. Note that we follow the notation in Lichtenberg and Lieberman[17]. Consider the Bunker Hamiltonian expressed in action-angle coordinates and focus on the resonance $m\dot{\theta}_1 = \dot{\theta}_3$ i.e., $m\Omega_1(\mathbf{J}) = \Omega_3(\mathbf{J})$. This can be done by performing a canonical transformation $(\boldsymbol{\theta}, \mathbf{J}) \to (\hat{\boldsymbol{\theta}}, \hat{\mathbf{J}})$ via the following generating function

$$F_2(\boldsymbol{\theta}, \hat{\mathbf{J}}) = (m\theta_1 - \theta_3)\hat{J}_1 + \theta_3\hat{J}_3 + \theta_2\hat{J}_2 \tag{23}$$

Transforming the original Hamiltonian using the identities

$$\mathbf{J} = \frac{\partial F_2}{\partial \boldsymbol{\theta}} \;\; ; \;\; \hat{\boldsymbol{\theta}} = \frac{\partial F_2}{\partial \hat{\mathbf{J}}} \tag{24}$$

and averaging over $\hat{\theta}_3$ we obtain a Hamiltonian

$$\bar{H}(\hat{\boldsymbol{\theta}}, \hat{\mathbf{J}}) = \bar{H}_0(\hat{\mathbf{J}}) + \varepsilon g_m^{(13)}(\hat{J}_1, \hat{J}_3) \sin\hat{\theta}_1 \tag{25}$$

corresponding to an integrable Hamiltonian that is relevant to the resonance of interest. In the above, the coefficients $g_m^{(13)}$ are known from the Morse action-angle transformation and the zeroth-order Hamiltonian is given by

$$\bar{H}_0(\hat{\mathbf{J}}) = (-\omega_3 + m\omega_1)\hat{J}_1 + \omega_2\hat{J}_2 + \omega_3\hat{J}_3 - \frac{m^2\omega_1^2}{4D_1}\hat{J}_1^2 - \frac{\omega_2^2}{4D_2}\hat{J}_2^2 \tag{26}$$

Since the averaged Hamiltonian is ignorable in $\hat{\theta}_2$ and $\hat{\theta}_3$, the conjugate actions $\hat{J}_2 = J_2$ and $\hat{J}_3 = (J_1/m) + J_3$ (polyad for the specific resonance) are conserved. As usual, one now expands the above Hamiltonian about the fixed points to obtain a pendulum like Hamiltonian

$$\Delta\hat{H}(\hat{\boldsymbol{\theta}}, \hat{\mathbf{J}}) = \frac{1}{2}G(\Delta\hat{J}_1)^2 - F\sin\hat{\theta}_1 \tag{27}$$

which has a full width $\hat{W} = 4(|F/G|)^{1/2}$. In the original variables the full width of the $m\Omega_1(\mathbf{J}) = \Omega_3(\mathbf{J})$ resonance is estimated as

$$W \equiv m\hat{W} = 4\left[\frac{2D_1\varepsilon}{\omega_1^2} g_m^{(13)}(\hat{J}_1^r, \hat{J}_3^r)\right]^{1/2} \equiv 4f_{m:1}^{1/2}\sqrt{\varepsilon} \tag{28}$$

where we have denoted

$$f_{m:1} \approx \left(\frac{2D_1 G_{13}^{(0)}}{\alpha_1 \omega_1 G_{11}^{(0)}} \right) \left[\frac{2(E - r_+ r_- D_1)}{G_{33}^{(0)}} \right]^{1/2} (1 - r_-) \left(\frac{r_-}{r_+} \right)^{m/2} \tag{29}$$

In the above expression $r_{\pm} \equiv 1 \pm (\omega_3/m\omega_1)$, α_1 is the exponent in the Morse oscillator potential function, and we have taken $J_2 = 0$ to estimate the value of J_3^r using the zeroth-order Hamiltonian.

A very crude estimate for the threshold coupling strength ε by invoking only the dominant 1:1 stretch-bend resonance is to set the full width of the resonance to be nearly the entire bound range of the action J_1 with $J_2 = 0$. In other words, using parameters appropriate for Bunker's model # 6 and at $E = 34$ kcal mol^{-1}, we set $W_{1:1} \approx 16.3\sqrt{\varepsilon} \sim 13$. This yields the threshold value to be $\varepsilon_{max} \sim 0.64$. There are two factors worth mentioning at this stage. Firstly, Chirikov analysis is independent of the angles i.e., the estimate just made is independent of the specific angle slice being chosen. For such large values of $\varepsilon_{\max}$ this is not correct. Secondly, it is well known that the critical ε value estimated using a Chirikov analysis[18] is not very accurate. In fact, typically, the actual values obtained from dynamics are about half that of the Chirikov estimate. However, this estimate can be made sharper and more accurate in a number of ways using the Greene residue theorem[19,20] or the renormalization approach[18,21]. We do not undertake this task here since the improved estimates will only reduce the $\varepsilon_{\max}$ value. Thus, as argued next, a more reasonable estimate is $\varepsilon_{\max} < 0.32$.

From the analysis of the zeroth-order Hamiltonian we estimate the resonant actions for the 1:1, 2:1, and 3:1 stretch bend-resonances to be around $J_1^r \sim 6.5, 10.8$, and 12.2 respectively. These resonances can be clearly identified in the Arnold web shown in Supplementary Fig. 4b. Denoting the resonant actions by $J_{m:1}^r$, the condition for the overlap of the prominent 1:1 with the higher order stretch bend resonances can be written as

$$\left[2f_{1:1}^{1/2} + 2f_{m:1}^{1/2} \right] \sqrt{\varepsilon} = \frac{2}{3} \left[J_{m:1}^r - J_{1:1}^r \right] \tag{30}$$

In the above we have invoked the so called "two-thirds rule"[22] for heuristically correcting the simple Chirikov estimate for the role of higher order resonances. Using the above criterion with values appropriate for Bunker's model # 6 at $E = 34$ kcal mol^{-1}, we find $\varepsilon \sim 0.05$ and 0.1 for $m = 2$ and 3 respectively. Comparing to the webs computed using the FLI approach, the estimates are quite good. Thus, large scale chaos is predicted, and confirmed by computations, to occur already by $\varepsilon \sim 0.2 - 0.3$.

7 Supplementary Fig. 9. Dependence of the Arnold web on θ_2 angle.

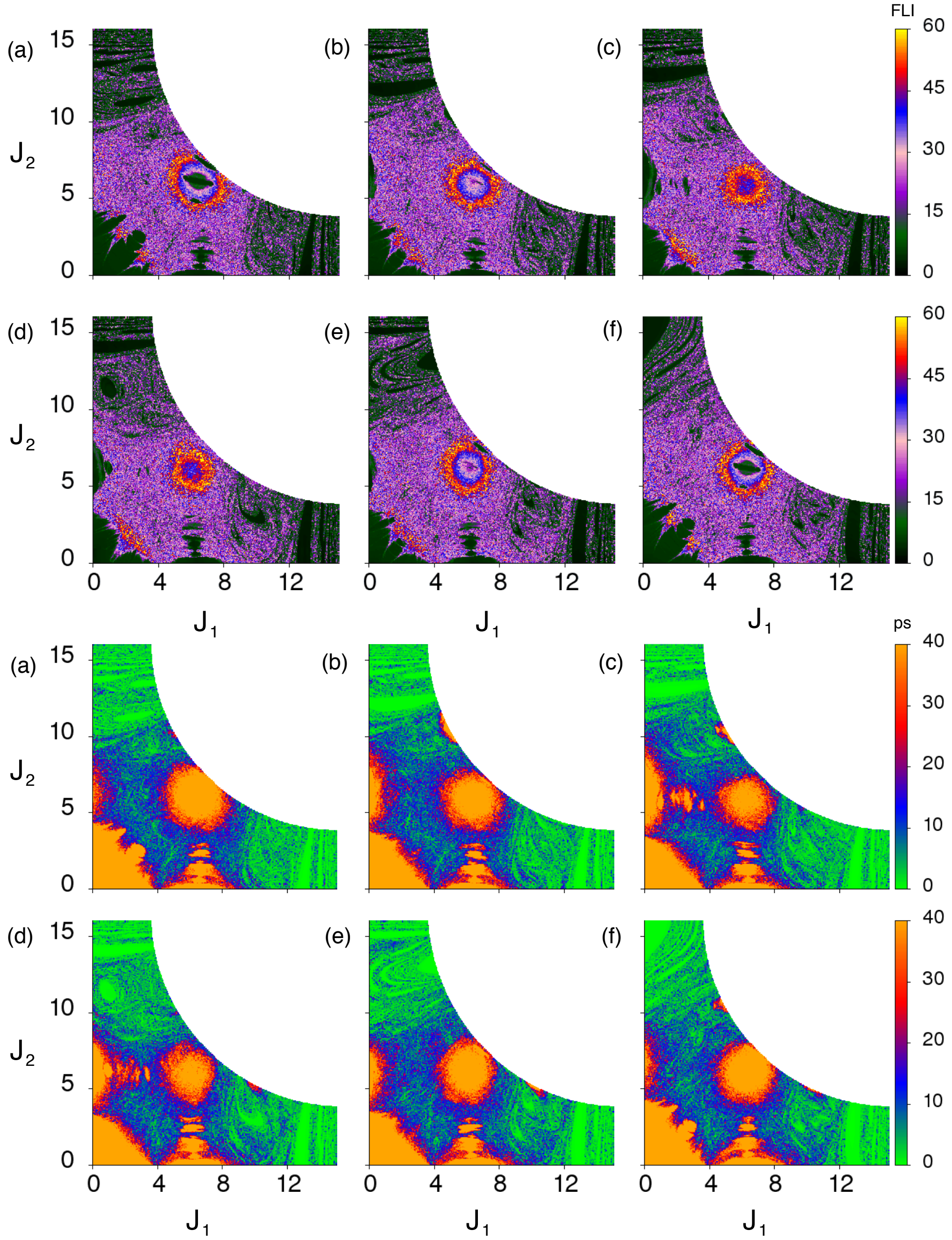

Supplementary Figure 9. Angle dependence of the web for model 6 at fixed $E = 34$ kcal mol^{-1} and $\varepsilon = 0.1$. Note that the web is shown over an extended range of FLI. The angles θ_1 and θ_3 are fixed at $\pi/2$ and 0 respectively. The angle θ_2 is varied as (a) 0 (b) $\pi/5$ (c) $2\pi/5$ (d) $3\pi/5$ (e) $4\pi/5$ (f) π. The lifetimes are shown in the bottom panel.

8 Supplementary Fig. 10. Dependence of the Arnold web on θ_1 angle.

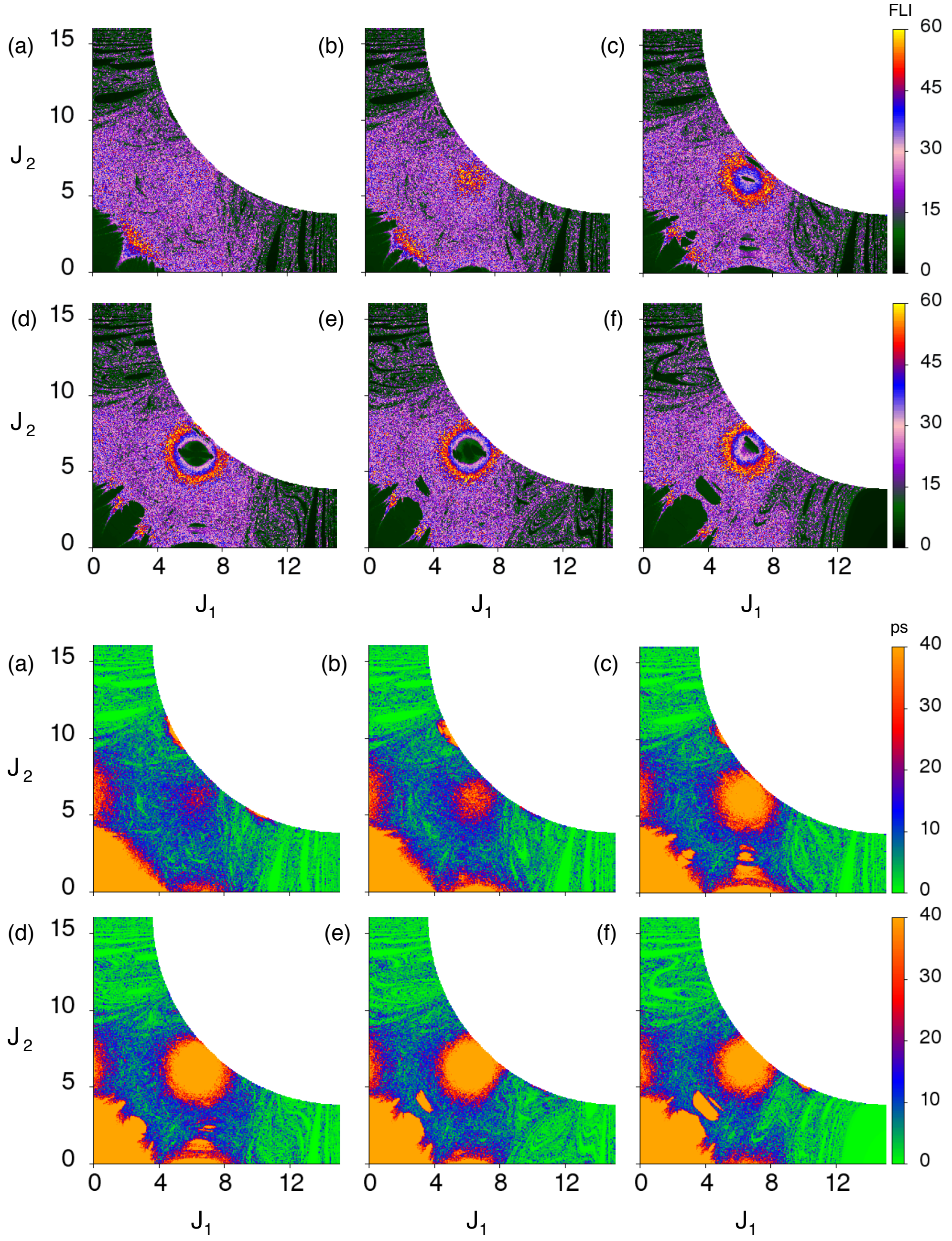

Supplementary Figure 10. As in Supplementary Fig. 9. Here the angles θ_2 and θ_3 are fixed at 0 and θ_1 is varied. (a) 0 (b) $\pi/5$ (c) $2\pi/5$ (d) $3\pi/5$ (e) $4\pi/5$ (f) π.

9 Supplementary Note 5

In the main text the trapping due to the $\mathscr{M}_{0,1,-1}^{1,0,-1}$ junction were highlighted. Nevertheless, the dynamics for other intermediate, but beyond the Chirikov overlap regime, values of ε exhibits trapping due to several other junctions. Here we provide some examples of such trapped intramolecular motion. In Supplementary Fig. 11 and Supplementary Fig. 12 we show the time-frequency analysis and the action space projection of the example long lifetime trajectories for ε = 0.2 and 0.3. Note that the action space projections are shown on a specific angle slice for convenience. In particular, the various actions at different times do not correspond to the initial angle slice. For example, the trajectory in Supplementary Fig. 11a is initially trapped in the vicinity of $\mathscr{M}_{0,2,-3}^{2,0,-3}$ junction for $\sim$ 5 ps followed by an equally long trapping near the $\mathscr{M}_{0,2,-3}^{1,0,-1}$ junction, before the eventual dissociation of the first stretching mode. The other examples are representative of different classes of trajectories that exhibit trapping near two or more junctions.

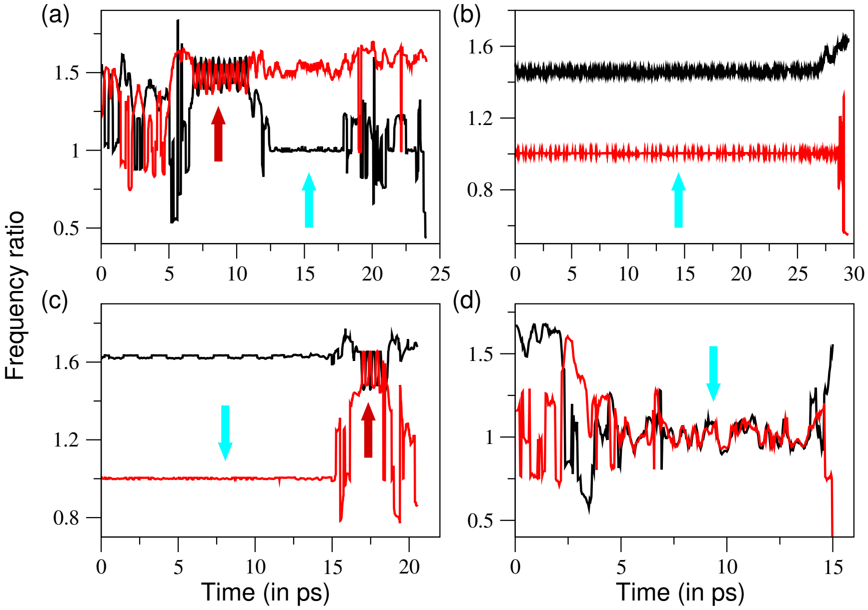

Supplementary Figure 11. Frequency ratios, $f_1 = \Omega_1/\Omega_3$ (black) and $f_2 = \Omega_2/\Omega_3$ (red) of representative long lifetime (t_{lt}) trajectories. Examples (a) $t_{\mathrm{lt}} \approx 24$ ps and (b) $t_{\mathrm{lt}} \approx 29.5$ ps are for $\varepsilon = 0.2$. Examples (c) $t_{\mathrm{lt}} \approx 21$ ps and (d) $t_{\mathrm{lt}} \approx 15$ ps are for $\varepsilon = 0.3$. The frequency ratios locking around particular rational (f_1, f_2) values indicates trapping of the trajectory near a resonance junction (shown in cyan and red arrows).

As mentioned in the main text, trajectories trapped at (f_1,f_2) junction in the FRS obey an approximately conserved polyad

$$P_d = f_1 J_1 + f_2 J_2 + J_3 \tag{31}$$

Note that P_d is conserved i.e., the Poisson bracket $\{H, P_d\} \approx 0$ for the duration that the trajectory is trapped near that specific junction. The same trajectory can then further get trapped near different junctions before dissociating. In Supplementary Fig. 12, the approximately conserved polyads are shown. Note that in Supplementary Fig. 12B, the correlated dynamics near $\mathscr{M}_{0,1,-1}^{1,0,-1}$ is established by the fact that the quantities $J_1 + J_3$ and $J_2 + J_3$ are not conserved in comparison to the full polyad.

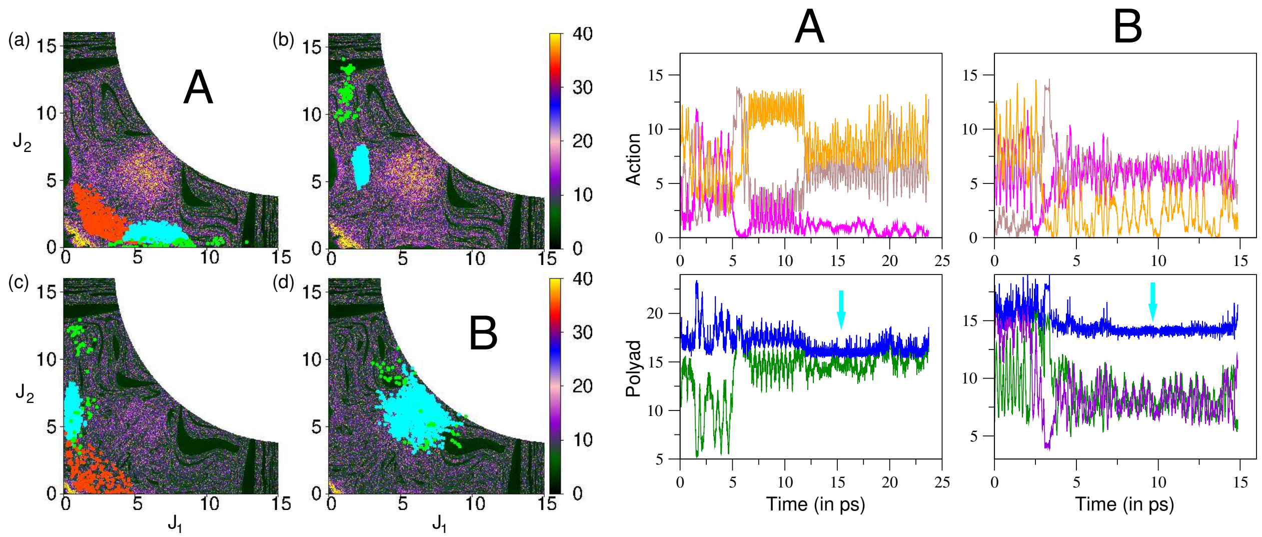

Supplementary Figure 12. Projection of actions, J_1 and J_2 on the Arnold web for the trajectories shown in Supplementary Fig. 11. Different trapping events near junctions are shown in cyan and red (as indicated in Supplementary Fig. 11). The portion of the trajectories shortly before dissociation are shown in green. Panels A and B show the variation of the zeroth order actions corresponding to the trajectories (a) and (d) respectively. J_1 (brown), J_2 (magenta) and J_3 (orange). Also shown are the appropriate approximately conserved polyads for trajectories A and B. The polyads (shown in blue) are $(J_1 + 3J_2/2 + J_3)$ for A and $(J_1 + J_2 + J_3)$ for B. Shown in green is the variation of $(J_1 + J_3)$ for both cases, while the violet line for B shows the variation of $(J_2 + J_3)$. The time period of polyad conservation is indicated by a cyan arrow.

10 Supplementary Note 6

The FRS density map in Fig. 4 of the main text indicates enhanced density near various junctions. For quantitatively determining the extent of locking near the junctions, we perform further analysis of the time-frequency data. The analysis is not straightforward since the resonances, and hence the junctions, are dense on the Arnold web. Strictly speaking, the width of a given resonance $\mathbf{r} \cdot \boldsymbol{\Omega} = 0$ of order $\mathscr{O}_r = |r_1| + |r_2| + |r_3|$ with $\mathbf{r} \equiv (r_1, r_2, r_3)$ is given by

$$\mathscr{W}_r \sim \sqrt{\varepsilon} e^{-\mathscr{O}_r} \tag{32}$$

Thus, at the intersection of two independent resonances of orders $\mathscr{O}_r$ and $\mathscr{O}_{r'}$ one needs to consider a $\mathscr{W}_r \times \mathscr{W}_r'$ rectangle in order to determine the trapping time statistics. Note that the size of the rectangular region also scales with the coupling strength ε. This approach, however, is not very practical since it requires a careful consideration of the total order, related to the timescales of dynamical interest, up to which resonances need to be considered. At the same time, for junctions involving large order resonances one requires significantly larger number of trajectories to obtain reliable statistics.

In this work we take a simple, but an approximate approach since we are interested in demonstrating that the trajectories spend time trapped in the vicinity of the junctions. In particular, we do not attempt here to do a detailed characterization of the trapped dynamics near junctions in terms of the crossing between specific order resonances. We have considered several key resonance junctions on the basis of number of visitations of trajectories in FRS density map

Supplementary Table 5. Key resonance junctions (f_1, f_2) considered (indicated by " ✓") to compute locking time distribution (Fig. 4e-f in the main text).

$\mathbf{f_1}$ (columns), **$\mathbf{f_2}$** (rows)

	**1/2**	**3/4**	**5/4**	**3/2**	**7/4**	**1/1**
**1/2**	✗	✗	✓	✓	✗	✓
**3/4**	✗	✗	✗	✓	✗	✗
**5/4**	✓	✗	✗	✗	✗	✓
**3/2**	✓	✓	✗	✓	✗	✓
**7/4**	✗	✗	✗	✗	✓	✓
**1/1**	✓	✗	✓	✓	✓	✓

and demarcated a square region around them with a tolerance $(f_1 \pm \delta, f_2 \pm \delta)$ with $\delta = 0.125$. In Supplementary Fig. 6, we show junctions $(1, 3/2)$, $(1, 1)$, and $(3/2, 1)$ as examples with the demarcated square region. The full list of the junctions considered in this work are shown in Supplementary Table 5. Note that too tiny a δ would not provide enough statistics and too large a value may include several nearby junctions. However, we did the calculations with a reasonably smaller δ value, and the results do not differ qualitatively.

11 Supplementary Fig. 13. Dependence of the Arnold web on energy.

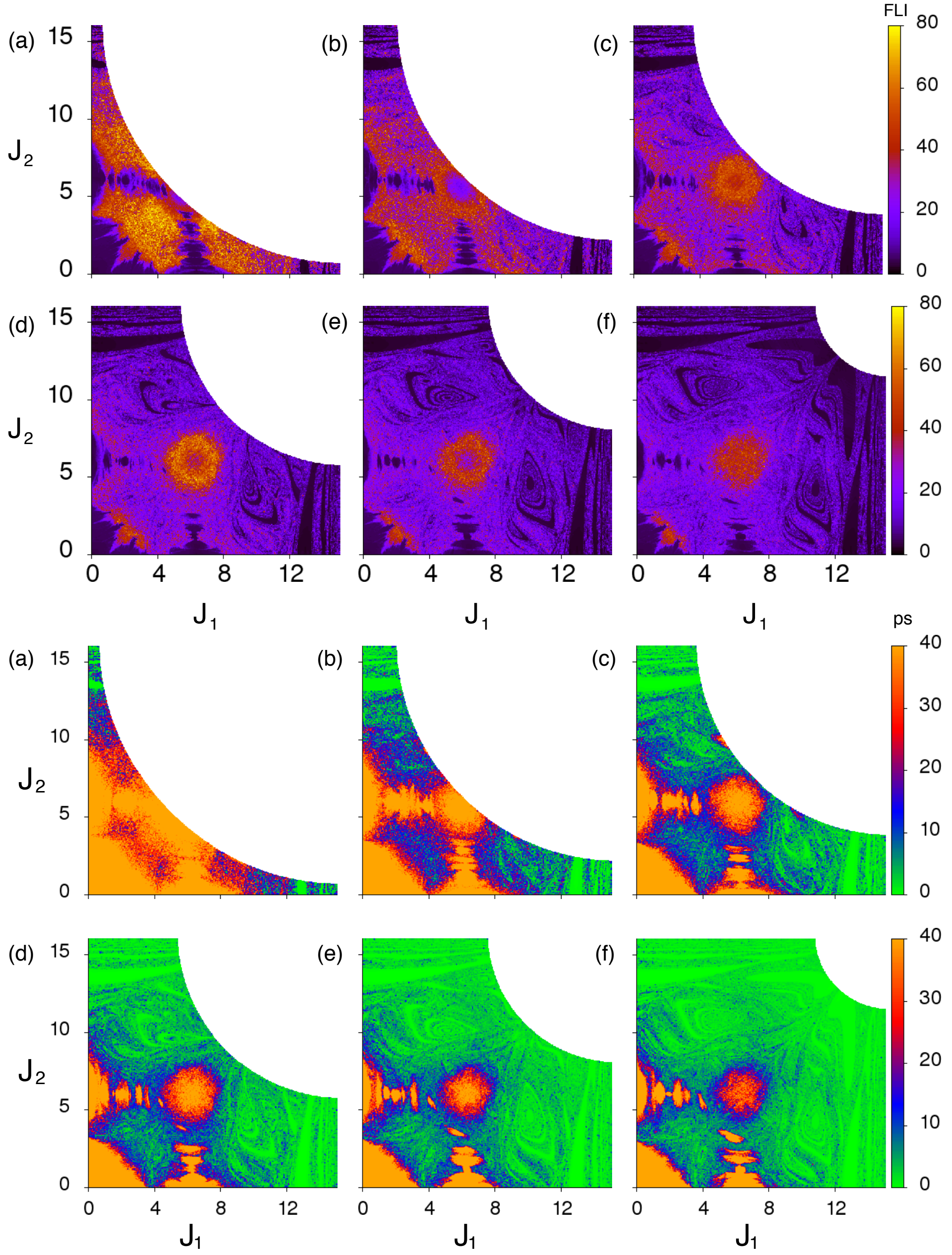

Supplementary Figure 13. Arnold webs for model 6 as a function of the total energy E for fixed $\varepsilon = 0.1$. (a) 26 kcal mol^{-1} (b) 30 kcal mol^{-1} (c) 34 kcal mol^{-1} (d) 38 kcal mol^{-1} (e) 42 kcal mol^{-1} (f) 46 kcal mol^{-1}. Corresponding lifetimes are shown in the bottom panel.

12 Supplementary Note 7

The model used in the current study uses the equilibrium G-matrix elements. This then allows us to make a detailed action-angle analysis for the system. For the original Bunker model, however, the action-angle transformations are not available. Therefore, an important issue is whether our results are generic enough. We believe there are reasons, apart from the fact that resonance junctions are ubiquitous in any nonlinear Hamiltonian system with $f \geq 3$, to expect the influence of the resonance junctions in a broader class of systems. For example, previous studies on the OCS molecule, with a Hamiltonian essentially of the form considered by Bunker, do indicate the presence of junctions that influence the IVR dynamics. It would be interesting to study the dissociation dynamics of OCS and link the deviations from RRKM behaviour with the extent of trapping near junctions. In addition, our preliminary studies on some of the other Bunker models suggest that the stabilisation near resonance junctions should be a key factor in understanding the origins of non-statistical reaction dynamics in more general systems as well. The purpose of this note is to summarize our preliminary results on a few of the other Bunker models and the full Bunker case for model 6.

- For reasons noted above, for the full Bunker case we cannot present the Arnold webs in the action space. However, the ZMS representation (Supplementary Note 3) can still be utilized and we expect that any indication of long lifetimes due to the influence of the resonance junctions should be discernible. Thus, the ZMS of the full Bunker model[23] with coordinate dependent G-matrix elements is shown in Supplementary Fig. 14a along with the corresponding lifetimes at $\varepsilon = 0.1$ and energy, $E = 34$ kcal mol^{-1}. Note that the nontrivial coupling due to $G_{33}(\mathbf{q})$ makes the system nonintegrable even at $\varepsilon = 0$. In particular, the ε values cannot be compared for the equilibrium G-matrix case and the coordinate dependent G-matrix case. Nevertheless, the wavelet time-frequency results shown in Supplementary Fig. 14 indicate the influence of resonance junctions in the system. One can clearly observe trapping near $\mathscr{M}^{1,0,-2}_{0,1,-1}$ junction and $\mathscr{M}^{1,0,-1}_{0,1,-1}$ junction. Note that for the full system as well the $\mathscr{M}^{1,0,-1}_{0,1,-1}$ junction is important.

- Here we present the web for two other Bunker models[24], model 8A and 8D. For both the cases, the equilibrium value of angle, $q_3^0 = \pi$ radians leads to $G_{i3}^{(0)} = 0$ $(i = 1,2)$, i.e. the system becomes an effectively two degrees of freedom system. We therefore artificially choose $q_3^0 = 2.0$ radians, resulting in three degrees of freedom models. The artificial value of q_3^0 can be thought of as the case wherein the molecule is in some excited electronic state with a bent geometry. Both the system have been studied at a total energy $E = 70$ kcal mol^{-1}. At this energy, the second stretching mode dissociates (dissociation energy, $D_2 = 60$ kcal mol^{-1}). We note that the original versions of these models were shown to be non-RRKM by Bunker.

  The evolution of the web with increasing coupling strength ε for the two model are shown in Supplementary Fig. 15a-c. Although the webs indicate the appearance of widespread chaos with increasing ε, note that a prominent junction around $(J_1, J_2) \approx (1.5, 7.0)$ (for model 8A) and $(J_1, J_2) \approx (1.0, 13.0)$ (for model 8D), indicated by arrows, persist for fairly large coupling strengths. The lifetime data for $\varepsilon = 1.0$ confirm that the junctions continue to influence the dissociation dynamics. An additional junction structure, with long lifetimes, can also be observed for model 8D.

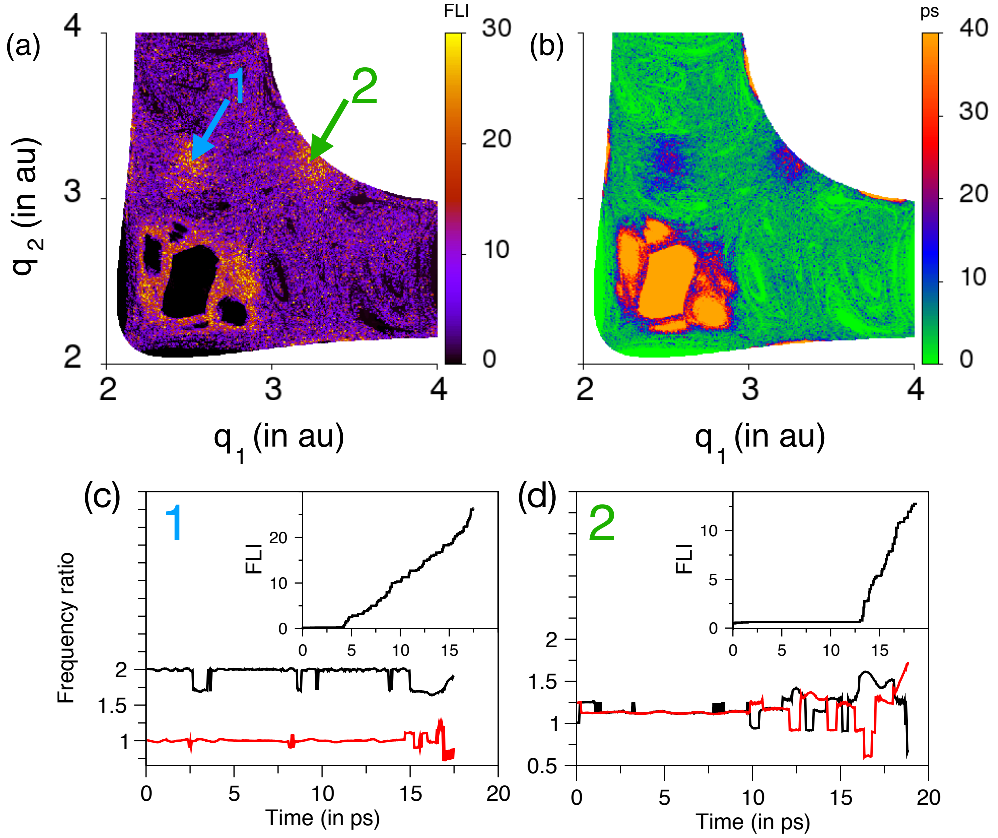

Supplementary Figure 14. (a) The ZMS and (b) corresponding lifetimes for the full Bunker model 6 at $E = 34$ kcal mol^{-1} and $\varepsilon = 0.1$. (c) and (d) Wavelet results for the two specific initial conditions indicated on the ZMS. These regions correspond to trapping near different junctions.

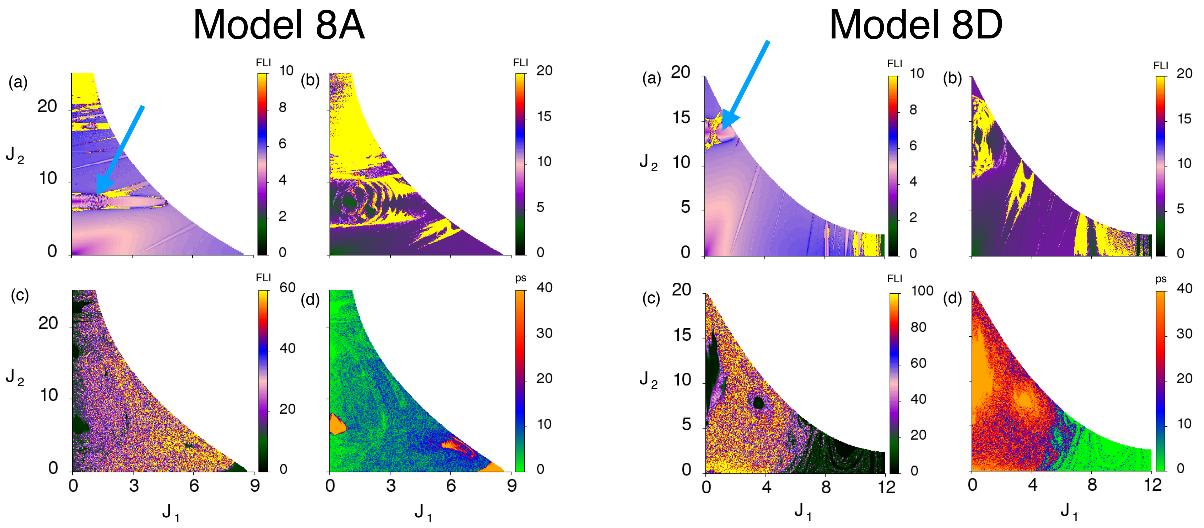

Supplementary Figure 15. The Arnold webs for models 8A and 8D at energy $E = 70$ kcal mol^{-1} and different ε values (a) 0.01 (b) 0.1 (c) 1.0. (d) Lifetime data for $\varepsilon = 1.0$. The angle slice is $(\theta_1, \theta_2, \theta_3) = (\pi/2, \pi/2, 0)$.

Supplementary References

1. Brankin, R. W., Gladwell, I. & Shampine, L. F. RKSUITE: A suite of Runge-Kutta codes for the initial value problem for ODEs. *SoftReport 91–S1* (1991).
2. Marcus, R. A., Hase, W. L. & Swamy, K. RRKM and non-RRKM behavior in chemical activation and related studies. *J. Phys. Chem.* **88**, 6717–6720, DOI: https://doi.org/10.1021/j150670a040 (1984).
3. MATLAB. *version 7.10.0 (R2010a)* (The MathWorks Inc., Natick, Massachusetts, 2010).
4. Skokos, C. H., Gottwald, G. A. & Laskar, J. *Chaos Detection and Predictability, Lecture Notes in Physics*, vol. 915 (Springer-Verlag Berlin Heidelberg, 2016).
5. Lega, E. & Froeschlé, C. On the relationship between fast Lyapunov indicator and periodic orbits for symplectic mappings. *Celest. Mech. Dyn. Astron.* **81**, 129–147, DOI: https://doi.org/10.1023/A:1013323507265 (2001).
6. Karmakar, S. & Keshavamurthy, S. Relevance of the resonance junctions on the Arnold web to dynamical tunneling and eigenstate delocalization. *J. Phys. Chem. A* **122**, 8636–8649, DOI: https://doi.org/10.1021/acs.jpca.8b08626 (2018).
7. Froeschlé, C., Lega, E. & Gonczi, R. Fast Lyapunov indicators. application to asteroidal motion. *Celest. Mech. Dyn. Astron.* **67**, 41–62, DOI: https://doi.org/10.1023/A:1008276418601 (1997).
8. Barrio, R., Borczyk, W. & Breiter, S. Spurious structures in chaos indicators maps. *Chaos, Solitons & Fractals* **40**, 1697 – 1714, DOI: https://doi.org/10.1016/j.chaos.2007.09.084 (2009).
9. Vela-Arevalo, L. V. & Wiggins, S. Time-frequency analysis of classical trajectories of polyatomic molecules. *Int. J. Bifurc. Chaos* **11**, 1359–1380, DOI: https://doi.org/10.1142/S0218127401002766 (2001).
10. Martens, C. C., Davis, M. J. & Ezra, G. S. Local frequency analysis of chaotic motion in multidimensional systems: energy transport and bottlenecks in planar ocs. *Chem. Phys. Lett.* **142**, 519 – 528, DOI: https://doi.org/10.1016/0009-2614(87)80655-3 (1987).
11. Chandre, C., Wiggins, S. & Uzer, T. Time–frequency analysis of chaotic systems. *Phys. D: Nonlinear Phenom.* **181**, 171 – 196, DOI: https://doi.org/10.1016/S0167-2789(03)00117-9 (2003).
12. Manikandan, P. & Keshavamurthy, S. Dynamical traps lead to the slowing down of intramolecular vibrational energy flow. *Proc. Natl. Acad. Sci.* **111**, 14354–14359, DOI: https://doi.org/10.1073/pnas.1406630111 (2014).
13. Shirts, R. B. Use of classical Fourier amplitudes as quantum matrix elements: a comparison of Morse oscillator Fourier coefficients with quantum matrix elements. *J. Phys. Chem.* **91**, 2258–2267, DOI: https://doi.org/10.1021/j100293a011 (1987).
14. Shchekinova, E., Chandre, C., Lan, Y. & Uzer, T. Analyzing intramolecular dynamics by fast Lyapunov indicators. *J. Chem. Phys.* **121**, 3471–3477, DOI: https://doi.org/10.1063/1.1756875 (2004).
15. Chirikov, B. V. A universal instability of many-dimensional oscillator systems. *Phys. Reports* **52**, 263 – 379, DOI: https://doi.org/10.1016/0370-1573(79)90023-1 (1979).

16. Oxtoby, D. W. & Rice, S. A. Nonlinear resonance and stochasticity in intramolecular energy exchange. *The J. Chem. Phys.* **65**, 1676–1683, DOI: https://doi.org/10.1063/1.433301 (1976).

17. Lichtenberg, A. J. & Lieberman, M. A. *Regular and Chaotic Dynamics*, vol. 38 (Springer-Verlag New York, 1992).

18. Escande, D. F. Stochasticity in classical Hamiltonian systems: universal aspects. *Phys. Reports* **121**, 165–261, DOI: https://doi.org/10.1016/0370-1573(85)90019-5 (1985).

19. Greene, J. M. A method for determining a stochastic transition. *J. Math. Phys.* **20**, 1183–1201, DOI: https://doi.org/10.1063/1.524170 (1979).

20. McKay, R. S. Greene's residue criterion. *Nonlinearity* **5**, 161–187, DOI: https://doi.org/10.1088/0951-7715/5/1/007 (1992).

21. Chandre, C. & Jauslin, H. R. Renormalization-group analysis for the transition to chaos in Hamiltonian systems. *Phys. Reports* **365**, 1–64, DOI: https://doi.org/10.1016/S0370-1573(01)00094-1 (2002).

22. Howard, J. E. Stochastic ionization of hydrogen atoms in a circularly polarized microwave field. *Phys. Rev. A* **46**, 364–372, DOI: https://doi.org/10.1103/PhysRevA.46.364 (1992).

23. Bunker, D. L. Monte Carlo calculation of triatomic dissociation rates. I. N_2O and O_3. *J. Chem. Phys.* **37**, 393–403, DOI: https://doi.org/10.1063/1.1701333 (1962).

24. Bunker, D. L. Monte Carlo calculations. IV. further studies of unimolecular dissociation. *J. Chem. Phys.* **40**, 1946–1957, DOI: https://doi.org/10.1063/1.1725427 (1964).
